# Supplementary material for: Causal interplay between lactose intolerance and gut microbiota: a combined bidirectional Mendelian randomization and in vivo validation study
Source: Front Nutr. 2026 Jun 1;13:1803337. doi: 10.3389/fnut.2026.1803337 (PMC13265576; doi:10.3389/fnut.2026.1803337)
Supplement: Supplementary file 1 [file Data_Sheet_1.zip › supplementary materials/Forward/leave one out cross validation/ebi-a-GCST90027767.finngen_R12_E4_LACTONAS.pdf]

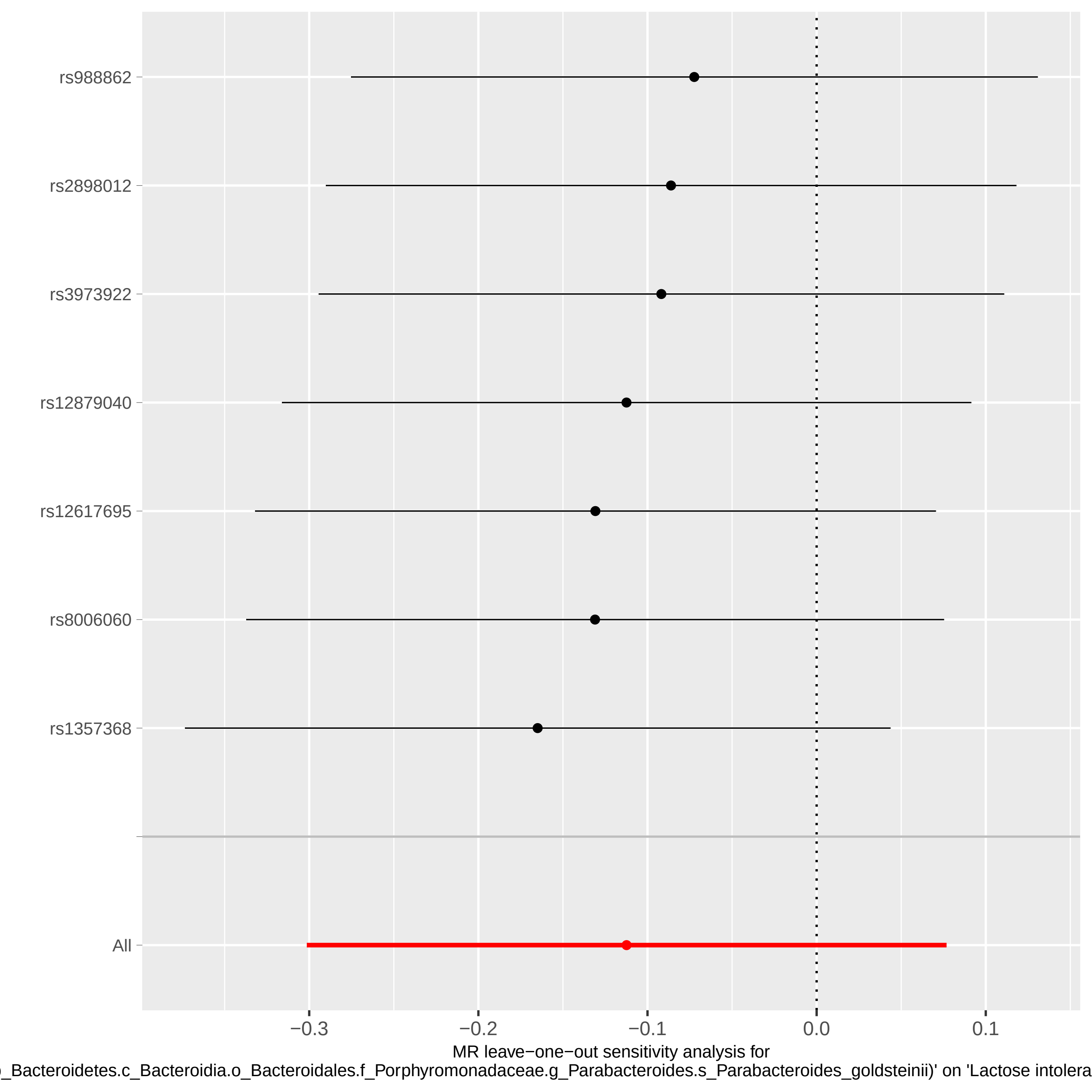

rs988862
rs2898012
rs3973922
rs12879040
rs12617695
rs8006060
rs1357368
All
−0.3
−0.2
−0.1
0.0
0.1
MR leave−one−out sensitivity analysis for
_Bacteroidetes.c_Bacteroidia.o_Bacteroidales.f_Porphyromonadaceae.g_Parabacteroides.s_Parabacteroides_goldsteinii)' on 'Lactose intolera
